# Trends in Organofluorine Chemistry Reveal Gaps in Knowledge on Environmental Persistence

**DOI:** 10.1021/acs.est.6c01045

**Published:** 2026-04-15

**Authors:** Carla A. Ng, Jacob F. King

**Affiliations:** Department of Civil & Environmental Engineering, 6614 University of Pittsburgh, Pittsburgh, Pennsylvania 15261, United States

**Keywords:** defluorination, emerging chemicals, pharmaceuticals, PFAS, energy, hazard assessment

## Abstract

The use of fluorination remains a common functionalization strategy across diverse fields of industrial and medicinal chemistry, but environmental fate studies have largely focused on perfluorinated compounds, leaving critical gaps in our understanding of how partially fluorinated substances degrade in the environment. This perspective discusses trends in modern organofluorine chemistry within the last five years and compares the structures proposed in those publications to those for which environmental fate studies exist. To facilitate comparisons, we define two simple metrics to describe fluorination of diverse chemical backbones: the fluorine atom fraction (FAF), and the presence of specific fluorinated groups, added through appending per- and polyfluorinated functional groups, such as −CF_3_ or −CF_2_H, to an existing chemical structure with a known functionality. Newly proposed substances have relatively lower degrees of fluorination than those that have been characterized for environmental degradation yet can achieve similar characteristics associated with “druggability”, such as lipophilicity and topological surface area. The extent of defluorination in the environment shows low correlation with FAF, though within subclasses of chemical backbones some trends emerge, highlighting the importance of the local bonding environment when fluorinated moieties are a small part of the overall structure of a chemical. Building a predictive understanding of how the type and degree of fluorination and backbone characteristics intersect to determine the environmental behavior of these compounds will be critical to enabling innovation while safeguarding environmental and human health.

## Graphical abstract

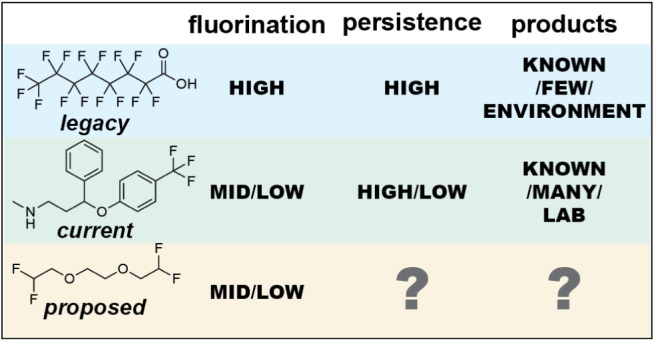

## 1 Introduction

The Stockholm Convention on Persistent Organic Pollutants,^1^ the most important international agreement focused on the control of hazardous substances, explicitly seeks to “protect human health and the environment from chemicals that remain intact in the environment for long periods.” Organohalogen compounds helped to define the concept of “persistence” as a hazard metric. The 12 initial persistent organic pollutants (POPs) listed in the Stockholm Convention are all organochlorine compounds, and the 16 “new POPs”, added between 2009 and 2023,^2^ introduced several additional organochlorine compounds, five types of brominated compounds (many of them flame retardants) and four types of per- and polyfluoroalkyl substances (PFAS), covering long-chain perfluoroalkyl carboxylic and sulfonic acids and their precursors.

The organochlorine compounds that helped launch environmental movements and regulatory frameworks on a global scale continue to be detected in even the most remote environments, decades after phase-out, as a result of their high persistence.^3^ In the meantime, PFAS are now understood to be much more persistent than other halogenated substances, leading them to be dubbed “forever chemicals.” PFAS helped to bring particular attention to persistence as a hazard metric, one that might even be used as a sole safety valve^4^ to prevent irreversible damage from as-yet-unknown toxic or dangerous effects. Perfluorinated alkyl acids have received intense attention from the research community to understand their environmental distribution and biological effects, as well as to devise engineered solutions^5–7^ to degrade them, given a paucity of natural degradation mechanisms, with few exceptions.^8–10^ Consumer concern^11^ and identified negative health impacts^12^ of several PFAS have yielded a mixture of voluntary phase-outs, regulatory actions, and proposed bans especially of long-chain perfluorinated substances.

Yet the development of new fluorinated chemicals continues apparently unabated, with applications across multiple industrial and consumer uses, including pesticides, pharmaceuticals, energy, electronics, and refrigeration. Over the past 10 years, 60% of approved pesticides have been fluorinated, half of which qualify as PFAS.^13^ Similarly, 23% of drugs approved by the US FDA between 2018–2022 were fluorinated,^14^ and in 2025, nearly 50% (14 out of 29) of small-molecule drugs approved by the FDA were fluorinated (about 30% of all drugs approved that year^15^). Clearly, fluorination is a key strategy of synthetic chemistry, often cited as a means to increase the biological half-life and bioavailability of pharmaceuticals, create high-efficiency refrigerants^16^ with lower ozone-depleting potential, withstand harsh conditions, promote low defect rates in electric and electronic components (solar cells, semiconductors^17^), and to increase the persistence and absorption of pesticides.^18^

Organofluorine chemistry thus remains an active area of research and development, supporting a specific journal (the Journal of Fluorine Chemistry), scientific society groups (e.g., the ACS Fluorine Chemistry Division), and conferences (e.g., the Winter Fluorine Conference). The strategies employed in the fluorination of molecules discussed in recent papers show both a move away from perfluorinated chains and an expansion of applications (e.g., into new bioactive compounds), with increasing use of fluorine-containing moieties (especially −CF_3_ groups, but also −CHF_2_ and −CH_2_F groups) or individual fluorine atoms added to a largely nonfluorinated backbone. In comparison to the large number of papers describing synthesis strategies and the performance of these semifluorinated compounds, there are few corresponding environmental fate and persistence studies published, particularly when considering the near-exponential growth of PFAS literature. Much of the environmental fate literature continues to focus on the well-studied perfluoroalkyl acids, but modern applications of fluorine chemistry may lead to different environmental behaviors and associated concerns. For example, the presence of trifluoroacetic acid (TFA) is increasing in the environment as a result of −CF_3_ group loss^19,20^ from fluorinated pharmaceuticals, pesticides, and refrigerants, among other sources. For other semifluorinated compounds, such as those without fully fluorinated carbons, the extent and result of environmental degradation are unclear. Biological defluorination of −CH_2_F and −CHF_2_ can occur, although rarely, due to the paucity of natural organofluorine substances to drive evolution.^21^

This perspective seeks to bring light on the emerging, less-studied area of semifluorinated compounds. The time between the commercial adoption of legacy PFAS in the 1940s and the publicly known environmental and human health impacts was ∼50 years.^22^ Unfortunately, the disconnect between industrial adoption and widespread acknowledgment of environmental effects is not unique to PFAS,^23^ and is in part an artifact of how chemicals are synthesized, introduced into the marketplace, and regulated. In an attempt to shorten the time window between chemical use and the investigation of impacts, we review the literature on the proposed uses and environmental fate of emerging fluorinated compounds to probe the following:

1) Are patterns emerging in their degree of fluorination, and are these patterns field-specific?
2) What is known about the environmental degradation of these substances?
3) What critical gaps remain that may hinder our ability to anticipate their environmental impact?

As part of our analysis, we specifically identify those chemicals that do not qualify as PFAS under the OECD definition:^24^ “fluorinated substances that contain at least one fully fluorinated methyl or methylene carbon atom (without any H/Cl/Br/I atom attached to it).” We used the identified PFAS and non-PFAS compounds to evaluate emerging trends. To aid in this analysis, we define several metrics for the degree of fluorination that consider both the addition of individual fluorinated moieties (e.g., CF_3_ groups) as well as the degree of overall fluorination of the molecule and discuss their uses and limitations.

## 2 Methods

### 2.1 Identification of Relevant Substances

We conducted two separate searches to get an overview of (i) the landscape of research on modern applications of fluorinated chemicals and (ii) what is known about their persistence in the environment.

For the first search, we identified studies via the Web of Science database using the following search terms: “fluorinat* AND (synthesis OR application OR strategy OR novel OR enable*)”. The search was constrained to the last six years and to publications in English only. This returned a total of 10,051 documents. These were then further refined by selecting the topic classes with at least 200 publications (Figure 1), resulting in 6,512 documents. The biggest category was “Synthesis” (based on Web of Science “meso” categories), followed by “Electrochemistry”. These 6,000+ articles were then manually curated as follows: papers were sorted by relevance, and 50 studies were selected from the top 150 results as follows: excluding articles that had only minimal mention of fluorinated products, inorganic fluorine compounds (e.g., some ionic liquids), polymers/resins (except for discussion of partially fluorinated precursor molecules for the synthesis of functionalized polymers), retrospectives reviewing synthesis routes from more than six years ago, purely *in silico* studies, and fluorination of amino acids within larger protein structures. Of the 50 publications selected in this fashion, chemical structures were extracted from those that had machine-interpretable names (40 of the 50 papers) that could be converted to a SMILES structure using the ChemDraw program (version 25.5.0.6237, Revvity Signals Software, Inc.). To minimize potential bias from this manual curation, we then further expanded the study database by resorting the 6,000+ articles by citation count and selecting the most cited papers (those with >200 citations from within the date range) that were neither polymers nor inorganic substances (14 studies) and from a further six papers from the initial relevance-sorted set selected at random to expand our total study database to 60 articles. Structures were manually extracted from these additional papers even if machine-readable names were not available by redrawing structures in study figures using ChemDraw.

**Figure 1:**
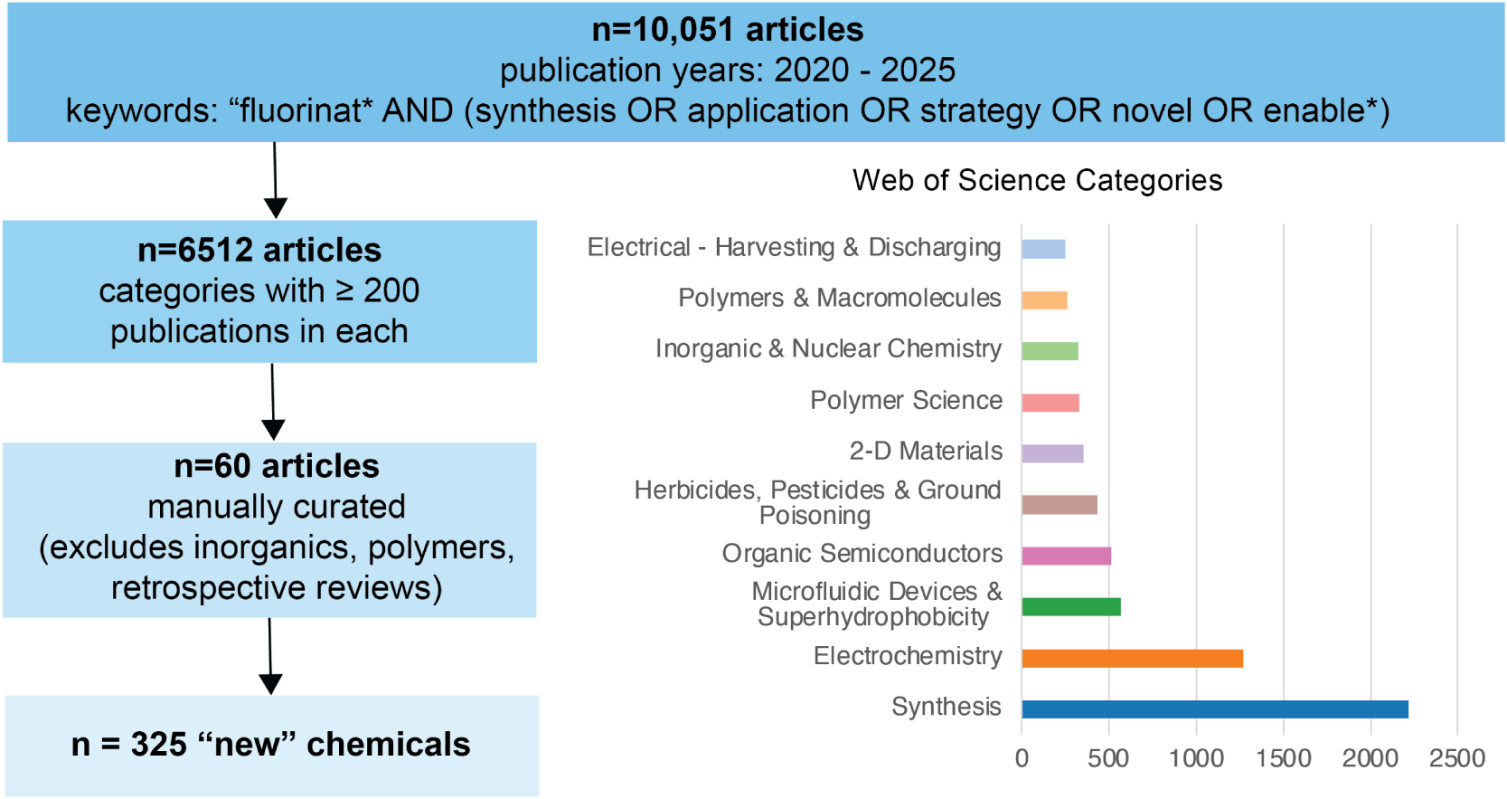
Search terms and curation of papers for the database of “new” chemical structures.

This yielded a database of >1,600 fluorinated chemical structures from 60 journal articles (Figure 1). Finally, the structures in this SMILES database were condensed to yield 325 representative substances (including at least one, and up to 12, structures per article). Representative structures were selected based on whether they were the focus of the study (e.g., fluorinated photocatalysts) or, in the case of broad “substrate scope” studies containing tens to hundreds of compounds, representatives for synthesis routes that were reported with the highest yields. The final set of extracted structures covered six broad use categories: pharmaceuticals and agrochemicals (combined into a “bioactive” category), energy (e.g., battery electrolytes), electronics/semiconductors (hereafter referred to as energy/electronics), and “other”, which included NMR tracers/chemical probes, industrial uses, catalysts, explosives, and polymer synthesis ingredients. The full spreadsheet of data with literature references for each molecule—referred to herein as “new” chemicals—is included in the Supporting Information (SI, Table S2).

For the second search, peer-reviewed studies published within the past 11 years (2015–2025) were identified using the Web of Science. A keyword search using the terms: (fluorinated AND (degradation OR transformation OR reactions) AND (environment* OR lake OR marine OR atmospher*)) returned 1,355 documents. Papers were filtered to remove those focused on engineered degradation systems (e.g., advanced oxidation processes and hydrated electrons). When parsing these papers for fluorinated molecules, well-studied highly fluorinated PFAS (e.g., perfluoroalkyl acids) were excluded except for a single representative of a specific structure (e.g., perfluorooctanoic acid was selected as the only perfluorocarboxylic acid). This yielded a database of 192 entries (with some limited substance repetitions in order to include additional fate characterizations, Figure 2).

**Figure 2:**
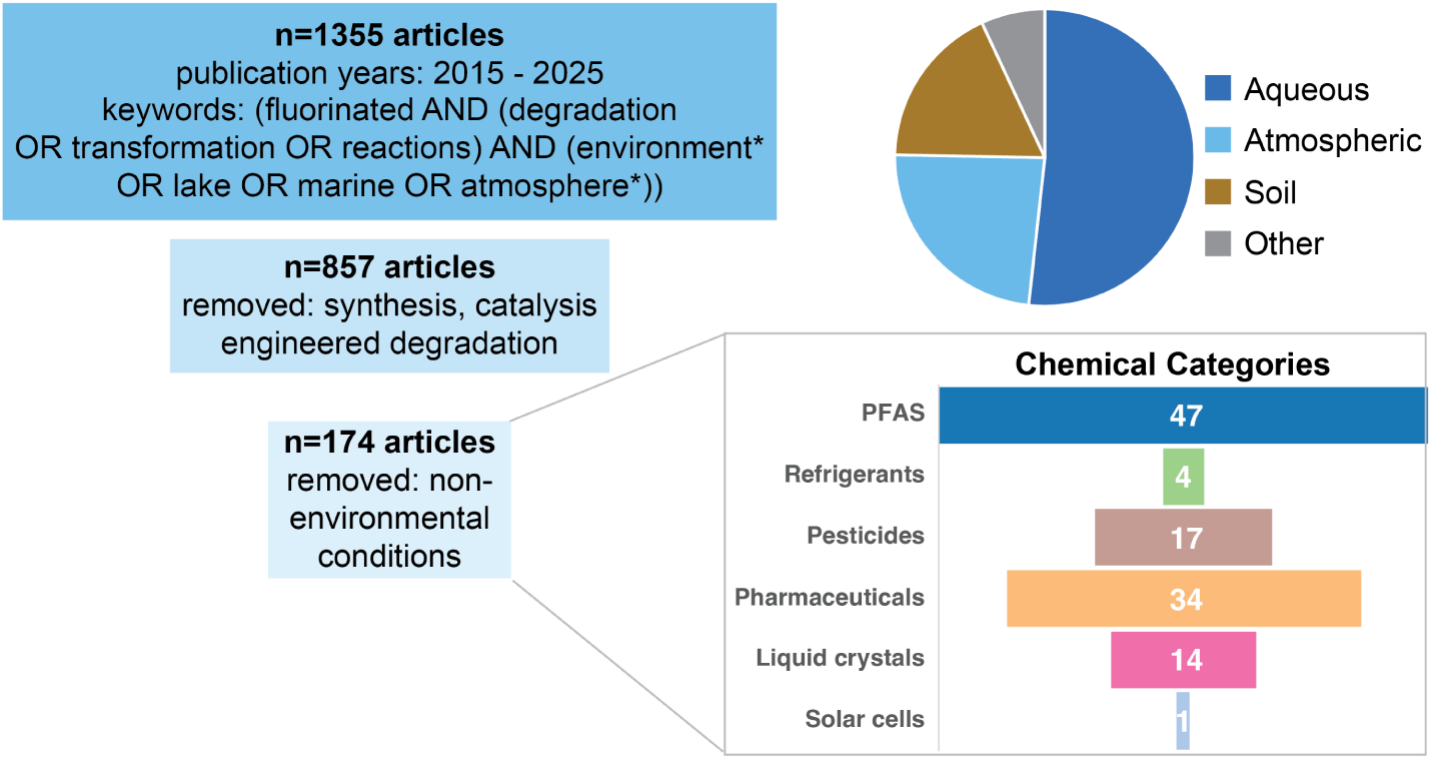
Bibliometric analysis of the scientific literature on the persistence and degradation of fluorinated molecules in the environment.

Their environmental transformation pathways were categorized as aerobic, anaerobic (biotransformation or on zerovalent metal particles), photolysis, or hydrolysis. Binary data (i.e., yes/no) were acquired on parent molecule removal, transformation product characterization, fluoride measurements, and closing the fluorine mass balance. Data on fluoride yield (*y_F_*, eq 1) were extracted as a quantitative measure of the environmental persistence of fluorine within the parent molecule, even if the parent molecule was removed. The full spreadsheet of data with literature references for each molecule—referred to herein as “characterized” chemicals—is included in SI Table S3.


$$y_{F}=\frac{\left[F^{-}\right]}{\left[Parent_{initial}-Parent_{final}\right]\times \left[F_{\mathrm{in parent}}\right]}$$ 1

### 2.2 Defining Degree of Fluorination

For the substances extracted through the two above searches, we defined two simple metrics to describe the extent (and method) of fluorination for each molecule that could be easily applied to a database of >500 structures. The fluorine atom fraction, FAF, is defined as (eq 2)­

2 $$\mathrm{FAF}=\frac{n_{F}}{n_{\mathrm{Tot}}}$$

where *n*_F_ is the number of fluorine atoms in the structure and *n*_Tot_ is the total number of atoms (including hydrogens). This overall degree of fluorination was then complemented by a second metric, the number of fluorine-containing groups (FG), which counts the number of fluorine-containing structures that were appended to an existing backbone, most commonly through the addition of one or more CF_*x*_, OCF_*x*_, or SCF_*x*_ groups. The FG was calculated as the sum of these groups on a molecule. To distinguish between direct fluorination of a backbone (captured by FAF), a −CF_3_ group was not counted toward the FG if it was terminal to an alkyl chain. Examples of structures and their respective FAF and FG values are provided below to further clarify how groups were identified separately from backbone fluorination. Finally, because enhanced lipophilicity and bioavailability are often cited as reasons for fluorination, we computed the log P, molecular weight (MW), and topological polar surface area^25^ (TPSA) for each structure using RDKit.^26^

### 2.3 Case Study—Impact of Fluorination Strategy on Predicted Lipophilicity and Persistence

To further explore the impact of specific fluorination strategies and structural backbones on properties key to chemical fate and potential hazards, we evaluated the impact of increasing degrees of fluorination on different backbone types and fluorination strategies with a small subset of 16 representative aromatic and aliphatic molecules (SI Table S1). For these molecules, we simulated the effects of (1) direct fluorination, (2) increasing fluorination of a methyl group (i.e., from CHF_2_ to CF_3_), and (3) increasing the number of fully saturated CF_2_ or CF_3_ groups, beginning from a nonfluorinated base case (benzene, toluene, trimethylbenzene, and butanoic acid).

For each resulting molecule, the log P of the molecule was calculated as described above using RDKit. The total bond dissociation energy (BDE), summed across all C–C, C–H, and C–F bonds, was calculated as a simple measure of the inherent persistence. Conformer searches were performed using RDKit with the ETKDGv32 algorithm to generate up to 10 conformers, which were subsequently optimized and ranked by MMFF94 energy.^27,28^ Conformers were further optimized with ORCA 6.0.16 ^29^ using B97M-V/def2-TZVPD.^30^ Finally, the lowest-energy conformer of each fragment and parent species was optimized, and frequency calculations were performed at ωB97X-V/def2-TZVPD.^31^

### 2.4 Statistical Analysis

Differences across groups for the new and characterized chemicals were evaluated for statistical significance by using a variety of methods. The Kruskal–Wallis test was used to compare the properties of the new compounds. P-values for *y*_*F*_ versus molecular metrics were calculated using one-way and two-way ANOVA. Linear regression was used to obtain slope, intercept, and R^2^ values when FAF was the independent variable for *y*_*F*_ evaluation. Finally, the Mann–Whitney U test was used to determine significant differences in comparisons between “new” and “characterized” chemical properties, and the Spearman correlation coefficient was calculated between FAF and MW, TPSA, and log P for these sets.

## 3 Results

### 3.1 Degree of Fluorination Metrics

The new and characterized fluorinated compounds identified through the two literature searches can be broadly classified, on the basis of what we will refer to as their “backbones”, as aliphatic, aromatic, or mixed (Figure 3). Within the aliphatic compounds, saturated, unsaturated, and cyclic compounds were identified. Similarly, the aromatic substances included several polyaromatic compounds, and both benzene and heterocyclic aromatics (e.g., pyridines and furans). As illustrated by the compounds used in Figure 3 to represent each category, their backbones can be quite complex, enabling multiple possibilities for fluorination either by directly adding fluorine to the backbone or by adding a fluorine-containing group.

**Figure 3:**
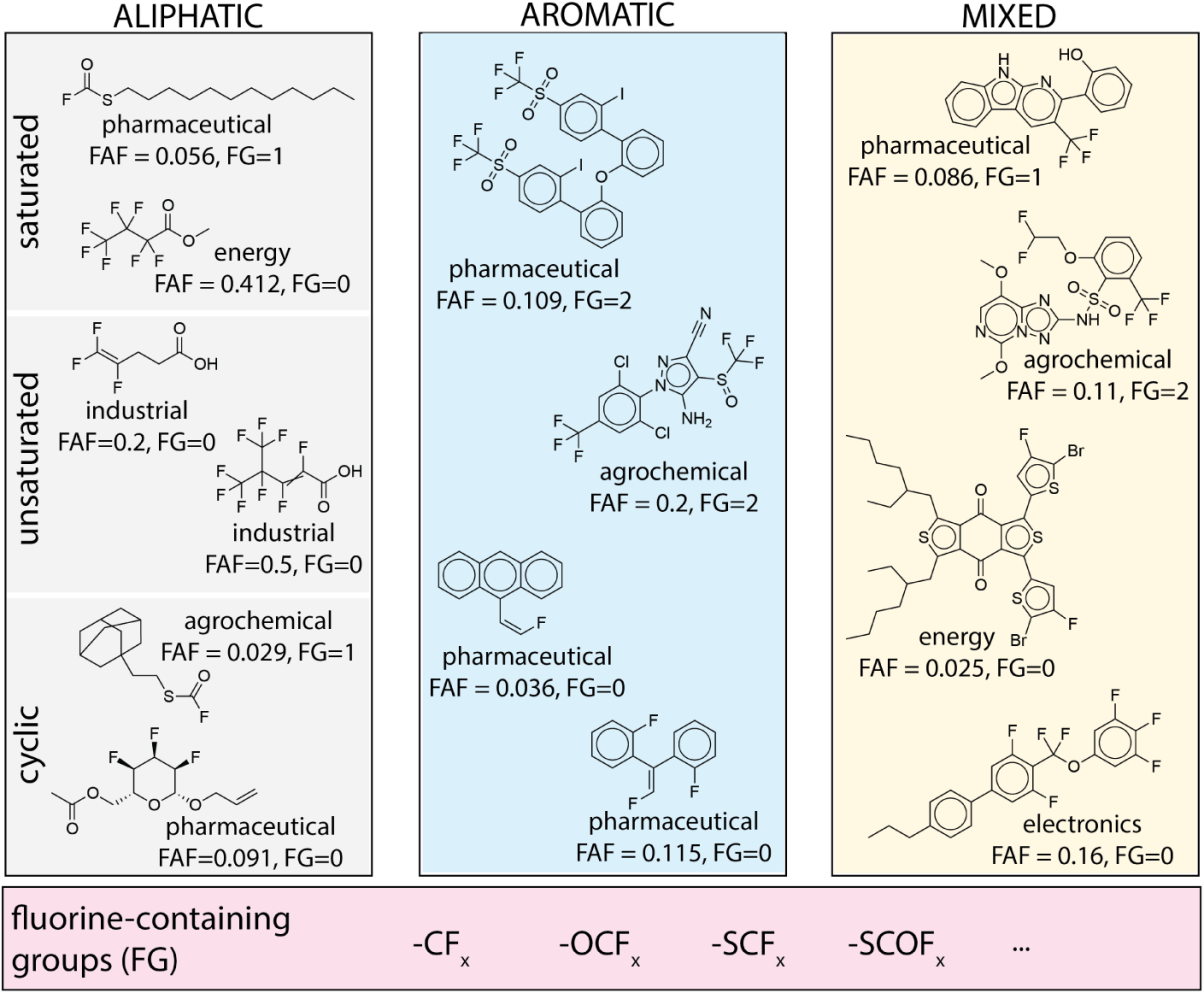
Examples of fluorination metrics for identified representative compounds. FAF: fluorine atom fraction; FG: sum of fluorine-containing groups.

Given this structural complexity, more refined fluorination metrics than FAF and fluorine group number and composition could be useful to separate differences in compound behavior that can be attributed to differences in the backbone and those that result from the degree, type, and position of fluorination. Such approaches can be difficult to implement systematically across structure types and thus are largely beyond the scope of this perspective. However, in Table 1 we suggest some benefits and limitations of a few such metrics that could be further developed in future work. Combinations of such metrics could also be devised to increase the coverage and exhaustiveness of compound descriptions.

**Table 1:** Fluorination Metrics Applied in This Work and/or Suggested for Further Development

			Compound Representation	
Proposed Metric	Benefit	Limitation	“New”	“Fate-Characterized”
FAF	Universal, easy to implement	No localization, does not account for backbone differences	100%	100%
Backbone-weighted FAF	More precise accounting for the ability to perfluorinate different structural motifs.	Difficult to generalize and apply across structure types	100%	100%
# F-groups −CF_*x*_, −OCF_*x*_, −SCF_*x*_	Key synthetic strategy that is an alternative to increasing FAF	Specific site matters, limited molecules, few data categories	28%	48%
F-group saturation	Easy to implement	Limited molecules, few data categories	28%	48%
ΔBDE^a^	Provides insight into potential fate (persistence) characteristics	Computationally expensive; may be subject to methodological weaknesses for fluorinated compounds	100%	100%
Δ log P^a^	Provides insight into potential fate and hazard characteristics	May not account for behavior of ionized compounds	100%	100%

a Calculated based on the molecule of interest compared with the defluorinated version, on a whole-molecule basis (e.g., the sum of BDE for all bonds).

### 3.2 Fluorination Strategy Case Study

We illustrate the interplay among the fluorination type, chemical backbone, and resulting chemical properties for a small representative subset of structures in Figure 4. Clearly, and as expected, both BDE and log P increase with increasing fluorination. However, interesting differences emerge within CF_*x*_ saturation (Figure 4B) and with the position of fluorination in the aliphatic butanoic acid structure (Figure 4D). For the fluorination of the methyl group in methylbenzene, there is a jump in log P between the mono- and difluoromethyl structure that is much larger than that between the difluoro- and trifluoromethylated forms (0.5 versus 0.09 log units, respectively). This “diminishing return” in lipophilicity is not echoed in the calculated BDE difference between adding two and three fluorine atoms (an increase of 39 versus 55 kcal/mol), suggesting that a benefit in greater lipophilicity can be realized with a smaller relative increase in persistence by employing −CF_2_ groups rather than −CF_3_. The reviewed literature on fluorination strategies for bioactive compounds highlights the unique properties of difluorinated molecules, particularly for bioactive compounds, which drives interest in the synthesis of difluorinated carbo- and heterocycles^32,33^ and carbonyls,^34^ among other medicinal chemistry building blocks.

**Figure 4:**
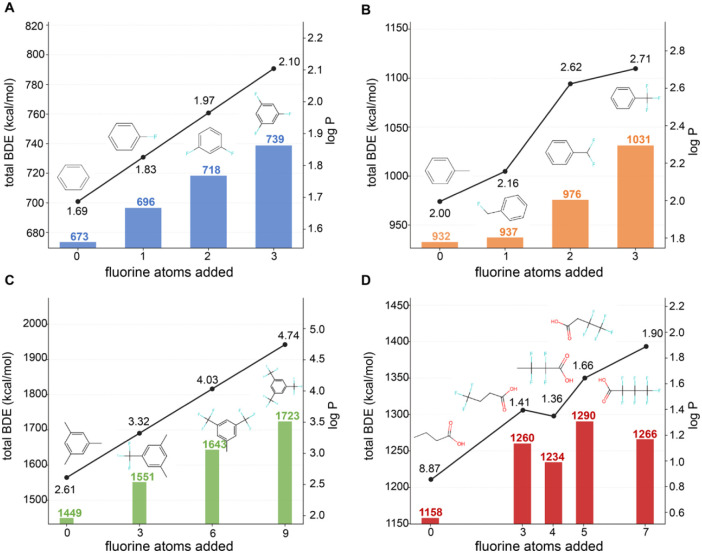
Effect of increasing fluorination on log P and total bond dissociation energy (BDE) by (A) directly fluorinating a simple aromatic structure (benzene); (B) increasing the degree of fluorine saturation on the single methyl group on toluene; (C) replacing methyl groups with fully fluorinated CF_3_ groups; and (D) fully fluorinating carbons along a butanoic acid alkyl chain.

For the single alkyl structure group explored in this case study (butanoic acid; Figure 4D), an interesting impact of the position of fluorinated groups emerges. Both log P and total BDE increase with the degree of fluorination except for the case where only one of the carbons in the alkyl tail is not fluorinated. Surprisingly, when the α-carbon (adjacent to the carboxylic headgroup) is a CH_2_, it has the highest total BDE among all the fluorinated structures, even the perfluorobutanoic acid (PFBA). This structure is distinct from most fluorotelomer substances, which typically have two or three CH_2_ groups proximal to the carboxylic acid head, not one, and are known to be amenable to at least partial degradation in the environment.^35,36^ When the nonfluorinated carbon is the terminal CH_3_ group, it has both the lowest log P and the lowest BDE of the fluorinated structures, highlighting the impact of the terminal CF_3_ (SI Figure S1). This has implications not only for the design of fluorinated alkyl substances but also for potential issues with degradation routes that may attack different positions along the alkyl chain. Of course, calculated BDEs only take into account thermodynamics, and cannot capture the influence of other important factors (e.g., steric hindrance, reaction intermediates) that can dictate overall degradation kinetics.

In the sections that follow, we explore the distribution of the simpler degree of fluorination metrics (FAF, number of F-groups, and their saturation) across a wider variety (>500) of substances proposed for application and those actually tested for environmental degradation, to better understand how much is known about the persistence of partially fluorinated substances and whether these metrics have any relationship to intended use, backbone type, or physicochemical properties.

### 3.3 Trends in Literature for Synthesis of New Compounds

The literature focused on the synthesis of new fluorinated compounds was dominated by bioactive compounds^32,34,37–44,44–61,61–79^ (Figure 5A). Single papers often discussed 50 or more different compounds, as “substrate scope” studies are common in medicinal chemistry. Such papers often focus on accessing specific structures (e.g., studies of chiral synthesis^56,80,81^ ), specific pathways,^39,51,51^ or even fluorinated photocatalysts.^82^ However, several papers identified in this search discussed fluorinated materials associated with energy production or storage (photovoltaics,^83,84^ batteries^69,85–87^ ) or energetic materials (explosives^88^).

**Figure 5:**
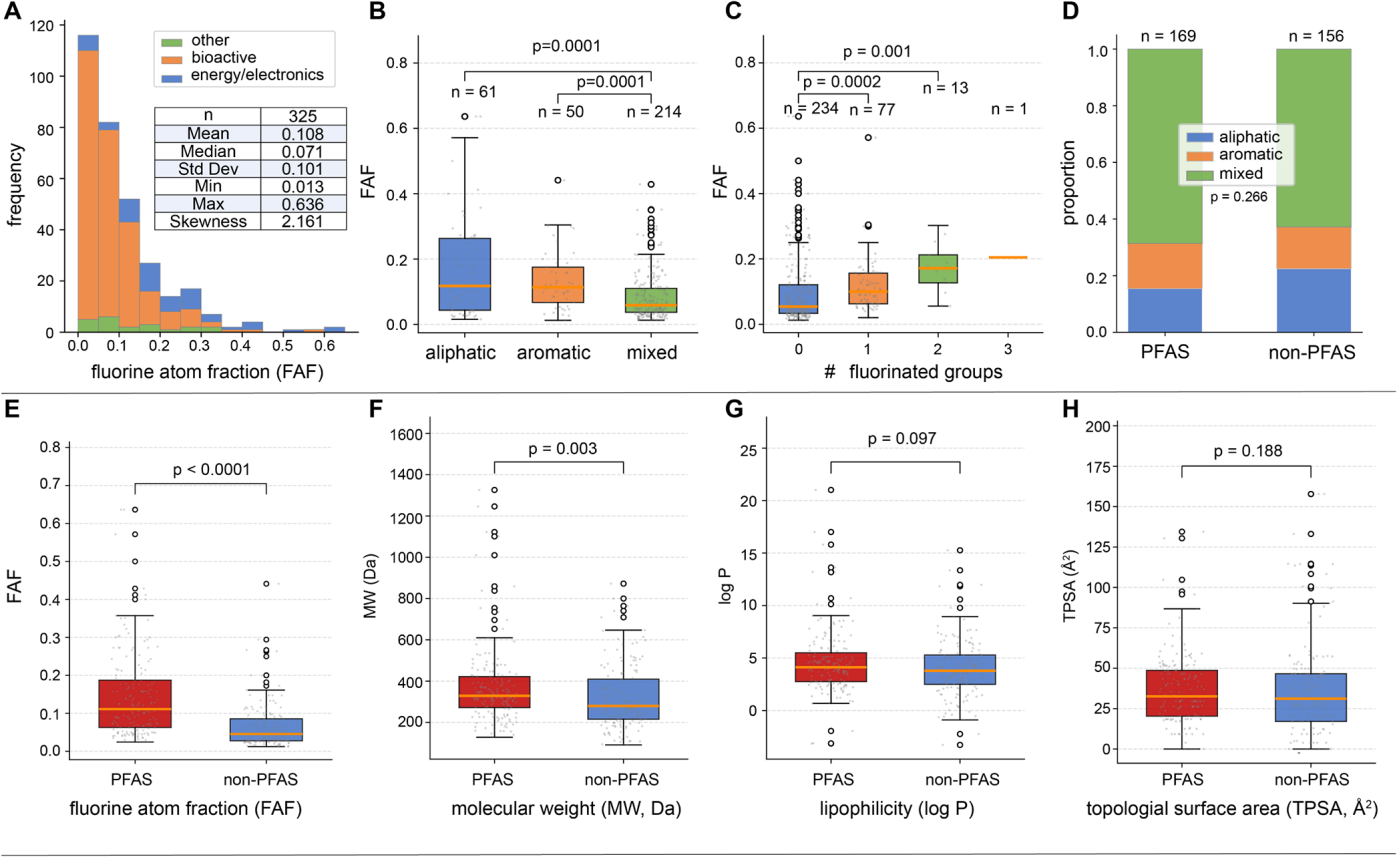
Characteristics of “new” compounds identified in the review of recent literature. (A) Fluorine atom fraction (FAF) distributions across use categories. (B, C) FAF distributions based on backbone and number of fluorinated groups. (D) Compounds were approximately evenly distributed between PFAS and non-PFAS compounds; a larger proportion of non-PFAS compounds with aliphatic backbones. (E) FAF and (F−H) physicochemical property distributions compared across PFAS and non-PFAS compounds.

Across the studies and potential applications, “mixed” backbone structures (those including both aromatic and aliphatic components) were most common (214/325 chemicals, or 66%, Figure 5B). There were small but statistically significant differences in the median FAF of these mixed backbone substances when compared to either aromatic or aliphatic compounds, which had slightly higher FAF. This is not entirely surprising, as the mixed backbone chemicals were generally larger and more complex, so the nonbackbone component would contribute more to the denominator of the overall fraction. The majority of substances (234/325, or 72%) had zero fluorinated groups, indicating only direct fluorination (Figure 5C), and the FAF of these substances covered the full range of values for the data set. The median FAF still increased significantly with an increasing number of FGs, likely because most of the fluorinated groups added to structures were fully fluorinated. However, only 14 substances had two or more FGs. Of the 325 extracted structures only 4% included a CF_2_H group, while 56 (17%) included only a single fully fluorinated methylene carbon (−CF_2_−). The maximum FAF among the 325 representative “new” chemicals was 0.636, for perfluoro 1,4-dimethoxybutane used in battery electrolyte solvents.^66^ The data set was approximately evenly split between PFAS and non-PFAS compounds, with slightly more of the PFAS subset consisting of mixed backbone structures (Figure 5D). Despite this approximately even distribution, the FAF was significantly higher among the PFAS compounds than among non-PFAS compounds (Figure 5E).

Given this difference in the degree of fluorination, it might be expected that the physicochemical properties differ significantly between the two subgroups. However, only molecular weight differed significantly, with the median molecular weight being slightly larger among the PFAS subset of the “new” chemicals (Figure 5F). Properties important to the “druggability” and environmental fate of these compounds, log P (Figure 5G) and TPSA (Figure 5H), showed no statistically significant differences between PFAS and non-PFAS compounds, indicating that backbone structure was more important than the degree of fluorination in determining these properties for most of the compounds (statistics summarized across groups in SI Figure S2). Thus, whether a compound qualified as a PFAS under the OECD definition did not substantially impact these properties. This can be seen as a two-edged sword—on the one hand, focusing only on PFAS compounds could miss other fluorinated molecules with similarly hazardous properties (e.g., bioaccumulation potential). On the other hand, it is also possible to achieve a desired level of lipophilicity and TPSA without high levels of fluorination, which might help moderate environmental persistence.

### 3.4 The Environmental Fate of Fluorinated Molecules

Fluorinated molecules that have been the focus of environmental fate and transformation studies fall into one of three categories: PFAS used in industrial applications, agrochemicals, and pharmaceuticals—although the fate of certain novel fluorinated chemicals (e.g., liquid crystal monomers)^89–91^ has been explored more recently. While the market share of commercially available bioactive molecules has been increasing, less information is available on the mass of specific chemicals used due to business confidentiality. Ongoing research is trying to piece together the contribution of specific industry sources to the total environmental organofluorine load.^92,93^ Even within specific uses, however, there can be extensive structural diversity of fluorinated molecules and overlap of structural archetypes between industries. By including molecules across all applications in fluorine fate analysis, we draw the most complete picture of how molecular properties and characterizations influence the fate of organofluorine in the environment.

There are several critical reviews that summarize PFAS,^94,95^ fluorinated agrochemical,^96^ and fluorinated pharmaceutical^97^ degradation in the environment. From these reviews and individual studies, 164 unique structures were identified, with representative studies included in Table 2 and the entire list of unique chemical and pathway combinations in Table S3. Some researchers tested a list of chemicals (up to >30) with minor structural variations, while others specifically examined a single parent molecule. Most structures had either partial or no perfluorination due to the lower probability of fully perfluorinated chemicals breaking down in the environment. A few representative fully perfluorinated molecules were included in Table S3 (e.g., perfluorooctanoic acid and perfluorooctanesulfonic acid), but these molecules were omitted from the degradation analyses to avoid negative bias in the results.

**Table 2:** Representative Environmental Fate Studies

Parent structures	# of chemicals	Uses	Backbone	Pathway	Environment	Degradate tracking methods^c^	Ref
Pharmaceuticals and pesticides (20), phenol derivatives (8), benzyl derivatives (8)	36	Bioactive	Aromatic, Mixed	Photolysis	Mercury vapor lamp, solar simulator, and specific wavelength LEDs	HRMS and ^19^F NMR for organics and F^–^	^98^ ^a^
Pharmaceuticals and pesticides (6), benzotrifluoride derivatives (22+)	28+	Bioactive	Aromatic	Photolysis	Xenon lamp	IC for F^–^	^99^ ^b^
Fluorinated carboxylic acids; linear, branched, unsaturated	13	Other (industrial/lubricant)	Aliphatic	Aerobic, anaerobic	Glass bottles inoculated with activated sludge and an unspecified anaerobic microbial community	HRMS for organics, FISE for F^–^	^100^
1-Fluoroalkanes (6), fluoroacetates (2), nonfluorinated analogs	8	Other (industrial/lubricant)	Aliphatic	Aerobic	Glass vials, *Pseudomonas* sp. Strain 273 isolated from garden soil	GC-MS, GC-HRMS for orgamics, IC for F^–^	^101^
Difluoro(3,4,5-trifluorophenoxy)methyl]-3, 5-difluoro-4′-propylbiphenyl	1	Energy/electronics	Mixed	Aerobic	Lab flask, soil culture enriched with BG1 (dehalogenation genes)	HRMS for organics, IC for F^–^	^91^

a Compilation of previous studies from the same research group.

b More derivatives were tested that did not show reactivity.

c Abbreviations: HRMS, high-resolution mass spectrometry; IC, ion chromatography; F-ISE, fluoride ion-selective electrode; GC-MS, gas chromatography—mass spectrometry; F^–^, fluoride.

From the larger list, 93 unique structures had information on defluorination (i.e., *y*_*F*_) during parent molecule degradation. Nine molecules were double-counted because multiple transformation pathways were investigated, and defluorination could differ by pathway (Figure 6B). Aerobic and anaerobic biotransformation pathways were exclusively studied for aliphatic molecules, whereas photolysis dominated for compounds that contained an aromatic moiety that absorbs light. Within each molecule class, however, differences in the *y*_*F*_ distribution versus the transformation pathway were limited. The molecular class breakdown of the 102 molecules (including double-counting) used in the fate analyses was 49 aliphatic, 31 aromatic, and 22 mixed. The median *y*_*F*_ of molecules in each class was in the order aliphatic < mixed < aromatic, although the data appeared bimodal, especially for aliphatic and mixed molecules (Figure 6C). The bimodal nature of the data suggests that once enough partial defluorination has occurred, additional defluorination becomes more feasible, likely because fluorine atoms protect molecules from degradation, and their absence leaves the molecule susceptible to degradation.

**Figure 6:**
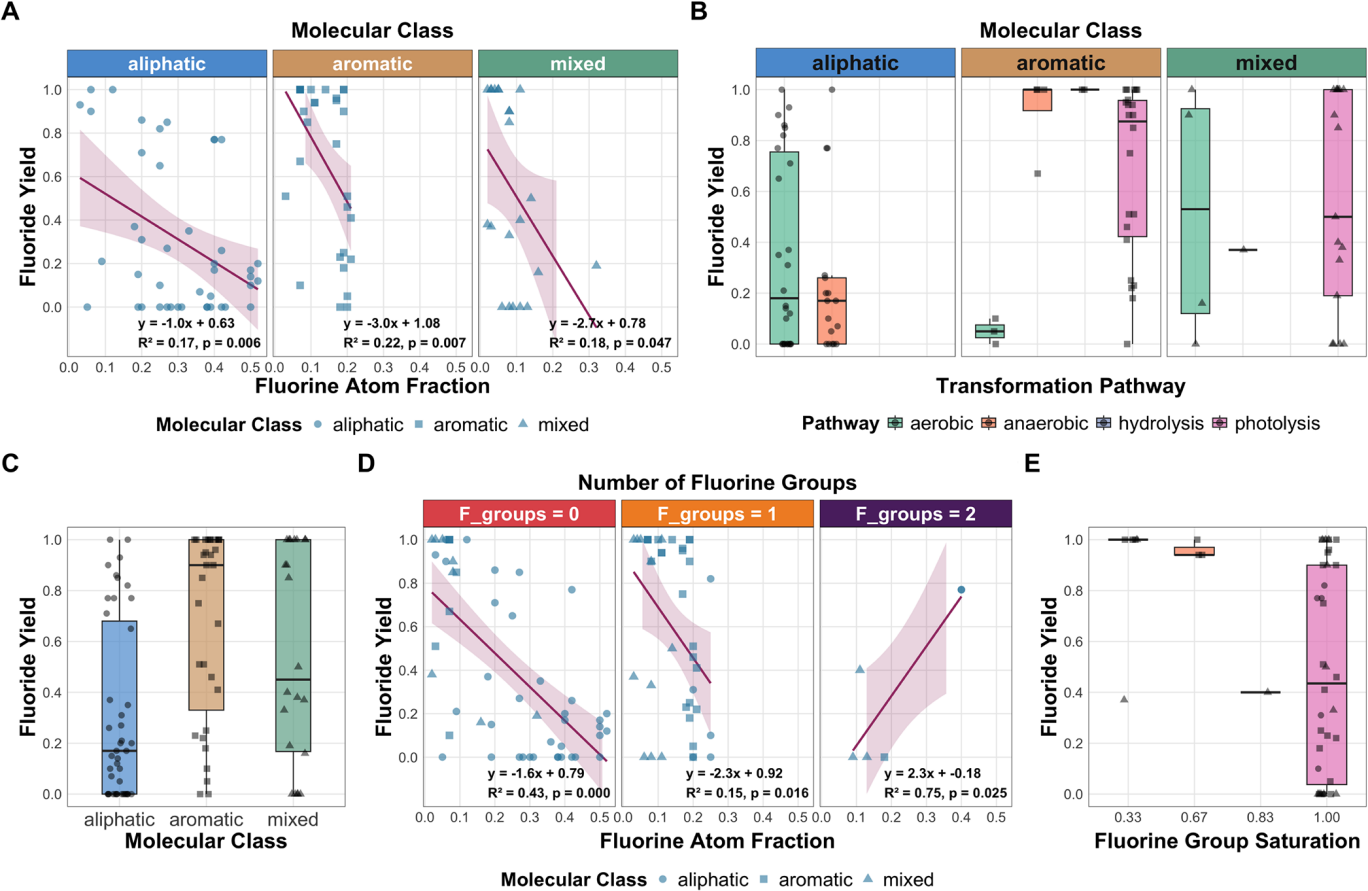
Fate of fluorinated molecules in the environment and correlations with molecular characteristics. (A) Relationships between fluorine atom fraction (FAF) and fluoride yield by molecular class. (B) Distribution of fluoride yield for different transformation pathways by molecular class. (C) Distribution of fluoride yield by molecular class for all transformation pathways. (D) Relationship between fluoride yield and FAF for substances with different numbers of fluorine groups. (E) Fluoride yield distributions for substances with different degrees of fluorine group saturation. P-values for box and whisker plots: (B) (two-way ANOVA) Conditions = 0.001, Class = 0.122, Conditions:Class = 0.0134; (C) (one-way ANOVA) Class = 0.0003; (E) (one-way ANOVA) Fluorine group saturation = 0.05.

Fluorine atom fraction (FAF) was an imperfect indicator by itself of *y*_*F*_ in each molecule class based on R^2^, although p-values were still significant (p < 0.05, Figure 6A). Examining FAF values, there were key differences in the FAF range by class. In aliphatic compounds, FAF ranged from 0.03 to ∼0.5, while its maximum for mixed and aromatic molecules was ∼0.3 and ∼0.2, respectively. Both mixed and aromatic compounds have benzene and heteroaromatic rings, where high degrees of fluorination are less likely. For example, no more than 4 fluorines were on a benzene ring, which corresponds to a FAF of 0.33 in the benzene moiety. The increased FAF range in aliphatics likely improved its p-value (∼0.006) with respect to *y*_*F*_. Slopes of the linear regressions were most negative for compounds containing aromatic moieties, although the values would likely be closer if a backbone-specific degree of fluorination was used in place of FAF (i.e., normalizing FAF to its maximum possible for each molecule, Table 1).

Beyond backbone classifications, several subclasses existed within each molecular class that influenced defluorination (Figure S3). For example, fluorine atoms could be attached to either saturated (sp^3^) or unsaturated (sp^2^) carbons in aliphatic compounds. While fluorine atoms near unsaturated carbons are easier to dissociate,^100^ both subclasses exhibited bimodal behavior (clusters around the median and ∼0.8), suggesting that the initial loss of fluorine does not always lead to greater defluorination. Differences in reactivity between subclasses were more pronounced for aromatic compounds, where fluorine or CF_*x*_ groups attached to a benzene correlated with greater *y*_*F*_ (median ∼ 0.9) than when attached to a heteroaromatic ring (median ∼ 0.4). Overall, the two-way ANOVA p-value associated with the effect of molecular subclass on *y*_*F*_ was <0.0001, compared to just 0.5 for the backbone class. Breaking molecules down into subclasses helps overcome resolution inadequacies when examining chemicals at the molecular class scale. For example, the Arnold group demonstrated that mean C–F bond dissociation enthalpies for −CF_3_ groups were lower when amended to benzenes versus heteroaromatics and appeared to correlate with *y*_*F*_.^98^ Along with bond dissociation energies, other molecular properties that influence defluorination could correlate with subclass, making the subclass a useful filter during the analysis of reactivity trends across broader molecular structures.

The number of fluorinated functional groups (FG) impacted the correlation between FAF and *y*_*F*_. The strongest correlation (R^2^ = 0.43) was observed for FG = 0; *y*_*F*_ decreased as FAF increased and its p-value was <0.001 (Figure 6D). When additional fluorine is incorporated into a molecule’s backbone structure, its potential to break down in the environment decreases. The negative correlation between *y*_*F*_ and FAF is also reflected by BDE values, where replacing a C–F bond with a C–H bond on a perfluorinated carbon adjacent to a PFAS headgroup decreases the sum of BDEs in the molecule (Figure 4D). For benzylic and aromatic molecules, *y*_*F*_ also correlates with BDE (Figure 4A–C); further investigation into BDE as a predictor of defluorination—and how to limit computationally expensive calculations when determining BDE—is a future research need. With regard to molecule synthesis, the strategy of adding individual C–F bonds to a molecule’s backbone to improve its utility directly increases its environmental persistence—and the amount of organic fluorine in the environment.

However, when a molecule had a single FG, its defluorination had significantly less dependence on FAF (R^2^ = 0.15). The wider range of FAF values for molecules with FG = 0 indicates that fluorine groups were generally added to compounds with a lower overall degree of fluorination. The lack of correlation between FAF and defluorination for FG = 1 suggests that other molecular properties play a more important role once fluorine groups are added to the molecule. For example, the degree of saturation of the FG (i.e., of the 3 possible fluorine positions in a single −CF_*x*_ group, what fraction is fluorine) influences defluorination. Groups containing 1/3 and 2/3 fluorine atoms were more likely to have higher degrees of defluorination (Figure 6E). Still, certain molecules that have an FG with 3/3 fluorine saturation experienced complete defluorination, implying that additional molecular properties are important, such as the specific structural environment where the FG is placed. The synthesis implications here are 2-fold. First, the high persistence of fluorinated chemicals with an inserted FG but low overall FAF allows chemical manufacturers to reduce the total fluorine content of molecules without sacrificing effectiveness, which decreases the flux of total fluorine into the environment. Second, chemicals may be synthesized by inserting an FG that renders them stable over their useful lifespan but ultimately breaks down to fluoride when exposed to environmental conditions, thereby minimizing organofluorine release, particularly using an FG that is not fully fluorinated.

### 3.5 Comparison of New and Fate-Characterized Chemicals

Given the relatively small number of studies on environmental fate compared to those on synthesis (hundreds compared to thousands) and the greater focus of the former on perfluorinated compounds, it is not surprising that the distributions of FAF for characterized and new chemicals differed substantially: the new compounds have a much greater skew toward low FAF, while the FAF distribution of the characterized compounds is broader and has a more pronounced right tail toward high FAF values (Figure 7A). Similarly, the frequency of compounds with one FG was similar to those with FG = 0 for the characterized compounds, whereas FG = 0 was much more common for the new substances (74% of representative new compounds; Figure 7A inset). When further resolved to separate the backbone classes (Figure 7B), it became clearer that this was driven in the characterized compounds by the aliphatic substances; these largely represent perfluorinated substances for industrial/lubricant uses (“other” category, Figure 7C).

**Figure 7:**
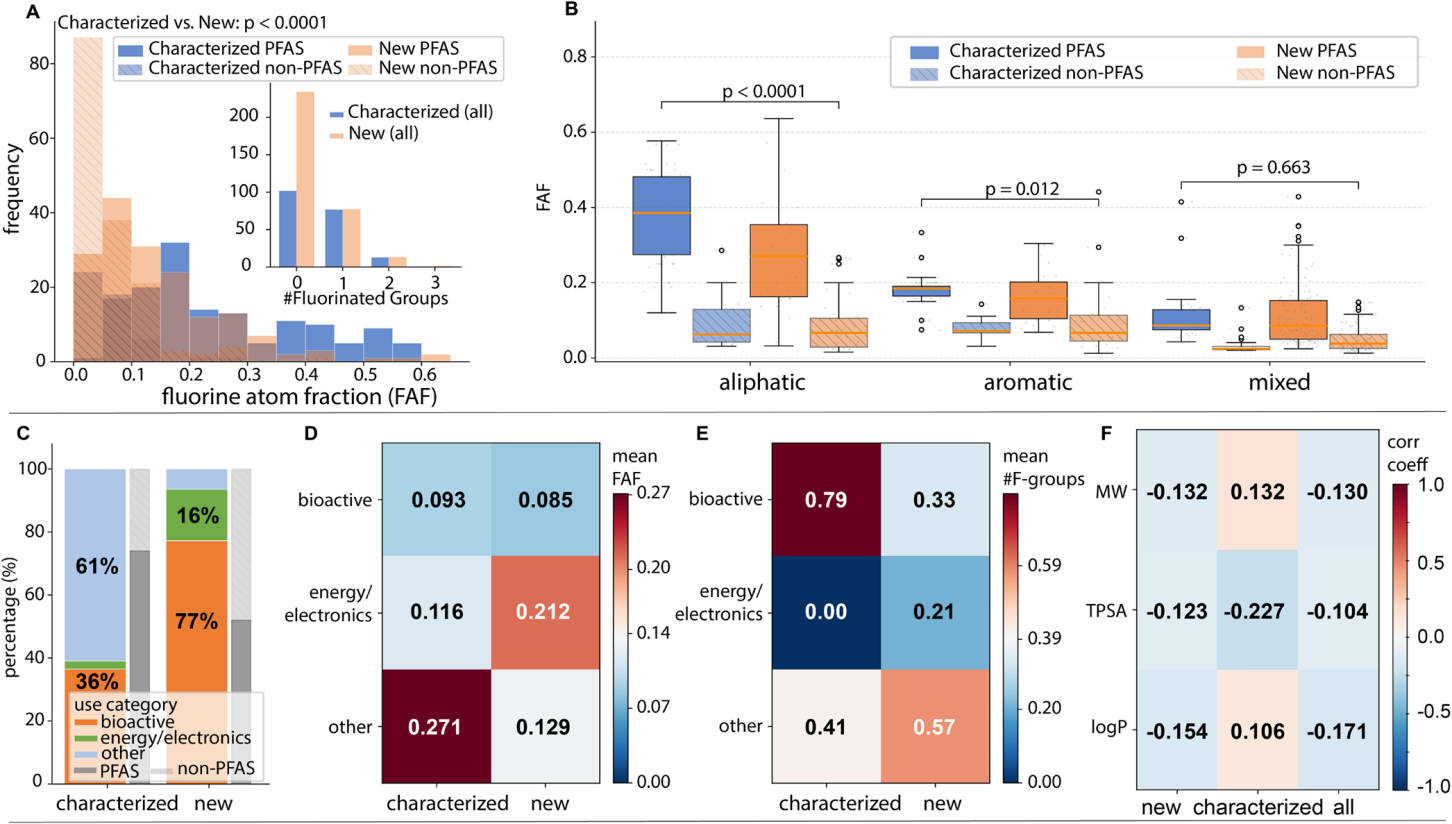
Comparison of “new” and “characterized” compounds by (A) fluorine atom fraction (FAF) and number of fluorinated groups across the full data sets; (B) separated by backbone type for PFAS and non-PFAS in the two groups; (C) comparing use categories. Correlations of (D) fluorine atom fraction and (E) number of fluorinated groups with use category. (F) Spearman correlation coefficient between physicochemical properties and FAF.

The differences in fluorination observed across the new and characterized compounds across use categories are further highlighted by higher average FAF values for the “other” category for characterized chemicals (Figure 7D), whereas energy/electronics applications have the highest FAF among the new chemicals. The “other” category yielded the highest average FG for new chemicals (0.57; Figure 7E) whereas, for the characterized substances, bioactive compounds had the highest average FG (0.79). Relatively few substances had FG > 1 among the new (4%) or characterized (5%) chemicals.

Finally, a comparison of FAF correlation with computed physicochemical properties yields important insight: relationships are weak overall but also substantially different between the new and characterized compounds. Based on the environmental fate analysis of characterized molecules, FAF by itself cannot predict defluorination but does show some relationship with the lipophilicity of the compound (driven by the highly fluorinated substances in the set). On the other hand, the new compounds show a slight negative correlation between FAF and physicochemical properties. This can be explained by the distribution of FAF across backbone types: the set is dominated by mixed-backbone substances with lower median FAF, whereas fewer more highly fluorinated substances are found within the aliphatic compounds, which have lower average molecular weights. Thus, lipophilicity, which increases not only with fluorination but also with overall molecule size, is decoupled from FAF in this set. An interesting further area of research would perform the same comparisons against nonfluorinated molecules with otherwise identical backbones, as was done for the smaller subset of representative molecules in Section 2, to better resolve the influence of fluorination on backbones with different levels of complexity and size.

### 3.6 Databases and Tools to Predict Environmental Transformation

Beyond critical reviews, publicly available tools including enviPath^102^ and the US EPA’s Chemical Transformation Simulator (CTS)^103^ provide insight into how molecules might transform in the environment. EnviPath details microbial degradation pathways, while CTS includes additional abiotic pathways, such as photolysis and hydrolysis. Both tools match structural groups within a molecule to libraries and databases with data pertaining to transformation schemes involving those groups, and both models are actively being trained on PFAS.

However, as the complexity of a molecule increases, the possible degradation pathways and transformation products increase exponentially. In addition, the tools may only show a certain number of transformation product generations, requiring additional queries with intermediates to find final products. As such, the use of these tools can be time-consuming and confusing for large molecules where the domain of possible products is extensive. To mitigate this complexity, both models incorporate the likelihood of specific transformation pathways in their outputs, and at least, the CTS includes a half-life ranking scheme for specific structural groups on the molecule. Continued development of such tools can generate data that would be helpful to integrate with metrics discussed here, with an eye toward improved frameworks for predicting the overall environmental persistence of compounds.^104,105^

### 3.7 Critical Gaps and Research Needs

Understanding which molecular properties drive the persistence of fluorinated chemicals in the environment is critical to inform future manufacturing that safeguards human and ecosystem health. Well-studied perfluorinated chemicals do not break down in the environment due to their unreactive carbon–fluorine backbones. However, a significant quantity of fluorinated but not perfluorinated chemicals continues to be introduced for a variety of uses. The environmental persistence of these molecules is much more variable and thus far has not been strongly correlated with specific molecular properties. Systematically varying molecular structures in experimental investigations is the first step in providing a basis for identifying which molecular properties most affect the environmental fate. Systematic structural variations have been applied to assess the fate of −CF_3_ containing benzenes^99^ and single heteroaromatic rings^106^ during photolysis. Photolysis conditions are simpler to replicate than microbial degradation conditions. However, systematic studies for microbial transformations of aliphatic compounds not amenable to photolysis will be a challenge. Standard protocols exist to assess biodegradability for general chemicals (e.g., OECD 301/308), but the specific conditions and organisms required to transform PFAS may not fit neatly into existing guidelines.

Furthermore, future studies should report on the fate of fluorine rather than focusing on parent molecule removal, due to the likely persistence of fluorinated transformation products. Many researchers are already acquiring data on organofluorine transformation products, and TFA is a widely cited example. Beyond TFA, there is value in finding other dead-end organofluorine product candidates. The challenge here is that the exact chemical formulas of these products will differ slightly based on minor parent molecule structural differences. One such strategy would be to accumulate candidate transformation products in a database and then sort them into subsets by molecular structure. Experiments to find these candidates should leverage emerging trends in fluorine synthesis rather than work with conventional PFAS, which have a limited number of possible transformation products. In particular, −CF_*x*_ (and to a lesser extent −OCF_*x*_ and −SCF_*x*_) groups can be added to commonly used structural backbones in different applications (e.g., benzenes, heteroaromatics, and fused rings in bioactive molecules). The goal would be to systematically alter the fluorine position and quantity to generate enough data to develop a machine learning model that can predict significantly more transformation products.

High-resolution mass spectrometry (HRMS) and ^19^F-NMR provide the capability to then find these predicted transformation products in complex environmental matrices. As evidence for this fluorine screening approach, Larsson et al. 2026 ^93^ accounted for ∼70% of the total extractable organic fluorine in an influent sample of municipal wastewater by referencing HRMS data to chemical libraries that contained known F-chemicals (e.g., conventional PFAS, bioactive molecules) and their commonly identified transformation products. However, organofluorine identification dropped to ∼40% in the treated effluent, likely due to the formation of unknown fluorinated transformation products. Following the above strategy to expand fluorinated transformation product databases could close this gap further.

Alongside environmental fate data, extensive characterization of new chemicals is necessary to provide an understanding of how these chemicals may behave in the environment. Performing fate studies for every new chemical is not feasible, but a robust model could ultimately replace the need for experimentation. A first step is generating data on a wide range of molecular properties, which can be accomplished by combining cheminformatics with thermodynamic properties (e.g., BDEs) computed by quantum mechanical modeling methods. One goal would be to resolve differences between how fluorination affects the properties and fate of aromatic versus alkyl compounds. In addition to thermodynamics, transformation kinetics impact the fate of molecules—especially if they only spend a finite time in specific environmental compartments. Metrics that capture kinetic effects—such as steric hindrances and local electron densities—should be included at the information-gathering stage. Generated data can then be inserted into computational models or artificial intelligence to find metrics that most affect the molecule’s fate. Knowing these metrics can allow for smarter molecule design that minimizes the level of organic fluorine accumulation in the environment.

Priorities pertaining to the environmental fate and synthetic utility of chemicals do not necessarily match. For example, environmental engineers and scientists can propose structural variations that have low environmental persistence, but those structures may not behave as required for a chemical’s use, whereas persistent structures may be difficult to replace. Enhanced communication between environmental scientists and synthetic chemists is critical to achieving highly functional and environmentally benign chemicals.

## Supplementary Material







## Notes

Figures S1 to S3 and Table S1 describing chemical structures and calculated properties for all collected new and characterized chemicals, statistical summaries of new chemical properties, and defluorination by subclass for characterized chemicals (PDF)
Table S2. Database of new compounds including SMILES, structure and structural sub-class, use category, fluorination metrics, and literature source (XLSX)
Table S3. Database of characterized compounds including SMILES, structure and structural sub-class, use category, fluorination metrics, degradation data, and literature source (XLSX)
