# Supplementary material for: Trends in Organofluorine Chemistry Reveal Gaps in Knowledge on Environmental Persistence
Source: Environ Sci Technol. 2026 Apr 15;60(16):11924–37. doi: 10.1021/acs.est.6c01045 (PMC13130954; doi:10.1021/acs.est.6c01045)
Supplement: Supplementary file 1 [file es6c01045_si_001.pdf]

Supporting Information For:

Trends in organofluorine chemistry reveal gaps in knowledge on environmental persistence

Carla A. Ng* and Jacob F. King

University of Pittsburgh, Department of Civil & Environmental Engineering

Table of Contents

Table S1. Case study compounds for impacts of fluorination on log P and BDE……….……S2

Figure S1. Bond dissociation energies for individual bonds in case study compounds………...S3

Figure S2. Statistical properties of new compounds……………………………………………S4

Table S2. New compounds database……………………………………………………………S4

Table S3. Characterized compounds database……………………………………………….S4

Figure S3. Defluorination by Sub-Class……………………………………………..............…..S5

Table S1. Case study compounds for the impact of fluorination on lipophilicity (as represented by logP) and persistence (as represented by total bond dissociation energies, BDE, in kcal/mol).

Base compound	Structure	SMILES	Total BDE (kcal/mol)	ΔBDE (kcal/mol)	logP	ΔlogP
**benzene**	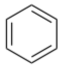	C1=CC=CC=C1	673.38	0	1.687	0
	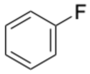	FC1=CC=CC=C1	696.43	23.05	1.826	0.139
	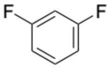	FC1=CC(F)=CC=C1	718.19	44.81	1.965	0.278
	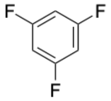	FC1=CC(F)=CC(F)=C1	738.6	65.22	2.104	0.417
**toluene**	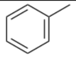	CC1=CC=CC=C1	932.44	0	1.995	0
	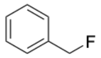	FCC1=CC=CC=C1	937.06	4.62	2.156	0.161
	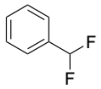	FC(F)C1=CC=CC=C1	975.51	43.07	2.624	0.629
	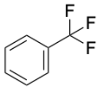	FC(F)(F)C1=CC=CC=C1	1031.01	98.57	2.705	0.71
**Trimethyl benzene**	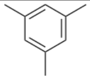	CC1=CC(C)=CC(C)=C1	1448.88	0	2.612	0
	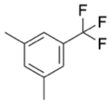	CC1=CC(C(F)(F)F)=CC(C)=C1	1550.92	102.04	3.322	0.71
	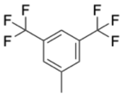	CC1=CC(C(F)(F)F)=CC(C(F)(F)F)=C1	1642.95	194.07	4.033	1.421
	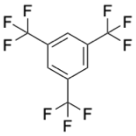	FC(F)(F)C1=CC(C(F)(F)F)=CC(C(F)(F)F)=C1	1723.17	274.29	4.743	2.131
**butanoic acid**	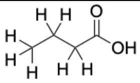	OC(C([H])([H])C([H])([H])C([H])([H])[H])=O	1157.58	0	0.871	0
	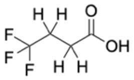	OC(C([H])([H])C([H])([H])C(F)(F)F)=O	1259.85	102.27	1.413	0.542
	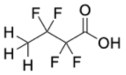	OC(C(F)(F)C(F)(F)C([H])([H])[H])=O	1234.27	76.69	1.362	0.491
	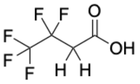	O=C(O)C([H])([H])C(F)(F)C(F)(F)F	1290.18	132.6	1.659	0.788
	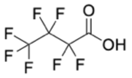	OC(C(F)(F)C(F)(F)C(F)(F)F)=O	1265.84	108.26	1.904	1.033

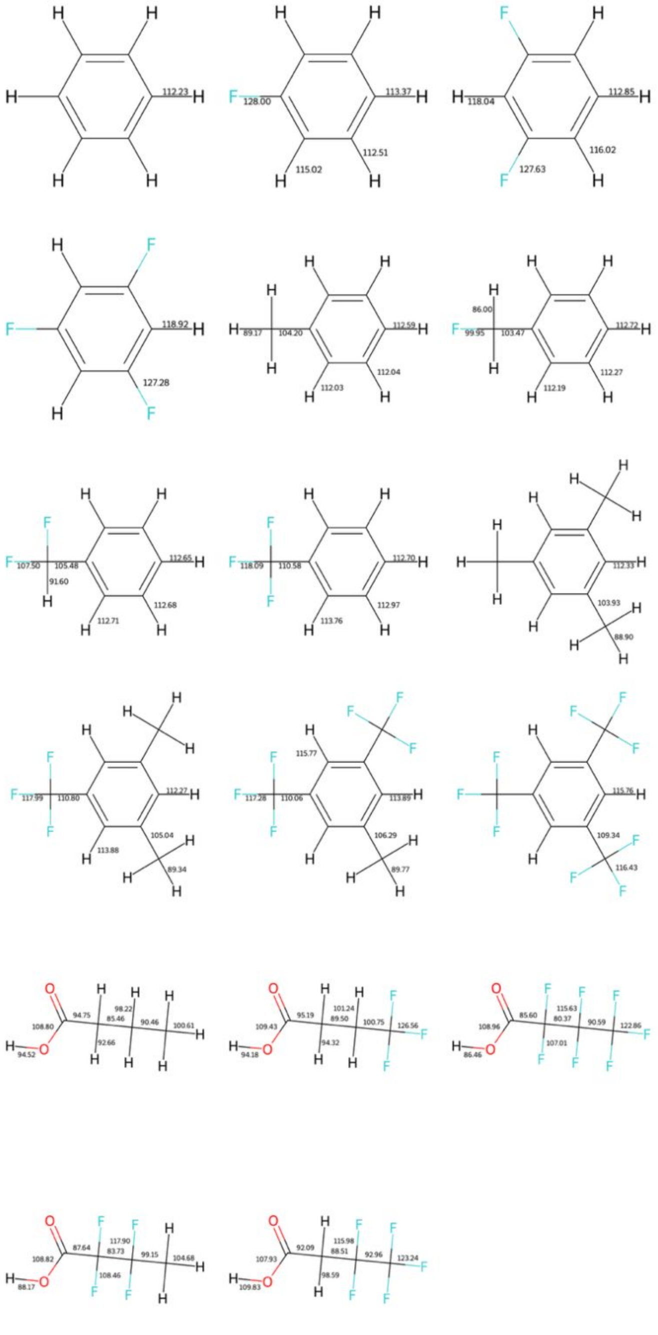

Figure S1. Bond dissociation energy (BDE) values for individual bonds in case study chemicals.

Group	Metric	n	Mean	Median	Std Dev	Min	Max	Skewness
All	Fluorine Atom Fraction (FAF)	325	0.108	0.071	0.101	0.013	0.636	2.161
All	MW	325	346.713	315.108	174.586	91.020	1326.008	2.096
All	logP	325	4.324	4.032	2.942	-3.280	21.019	1.427
All	TPSA	325	36.454	32.590	26.347	0.000	157.800	1.537
PFAS	Fluorine Atom Fraction (FAF)	169	0.147	0.111	0.115	0.024	0.636	1.764
PFAS	MW	169	370.254	329.159	188.426	128.009	1326.008	2.568
PFAS	logP	169	4.608	4.118	3.074	-3.148	21.019	1.835
PFAS	TPSA	169	36.719	32.590	22.079	0.000	134.500	1.302
Non-PFAS	Fluorine Atom Fraction (FAF)	156	0.066	0.045	0.061	0.013	0.441	2.738
Non-PFAS	MW	156	321.210	279.616	154.832	91.020	872.345	1.062
Non-PFAS	logP	156	4.016	3.784	2.768	-3.280	15.269	0.799
Non-PFAS	TPSA	156	36.168	31.145	30.373	0.000	157.800	1.582

Figure S2. Summary of statistical properties of new chemicals.

[**Table S2.** Database of new compounds. This table includes compound names, SMILES, structures, category of use, backbone type, and fluorination metrics (fluorine atom fraction and number and type of fluorinated groups). References for each compound are included as study doi. For the full table, please download Table S2.xlsx file.]

[**Table S3.** Database of characterized compounds. This table includes compound names, SMILES, structures, category of use, backbone type and sub-type, fluorination metrics (fluorine atom fraction, type, number, and saturation of fluorinated groups) and degradation metrics extracted from studies. Literature sources for each entry are included as study doi. For the full table, please download Table S3.xlsx file.]

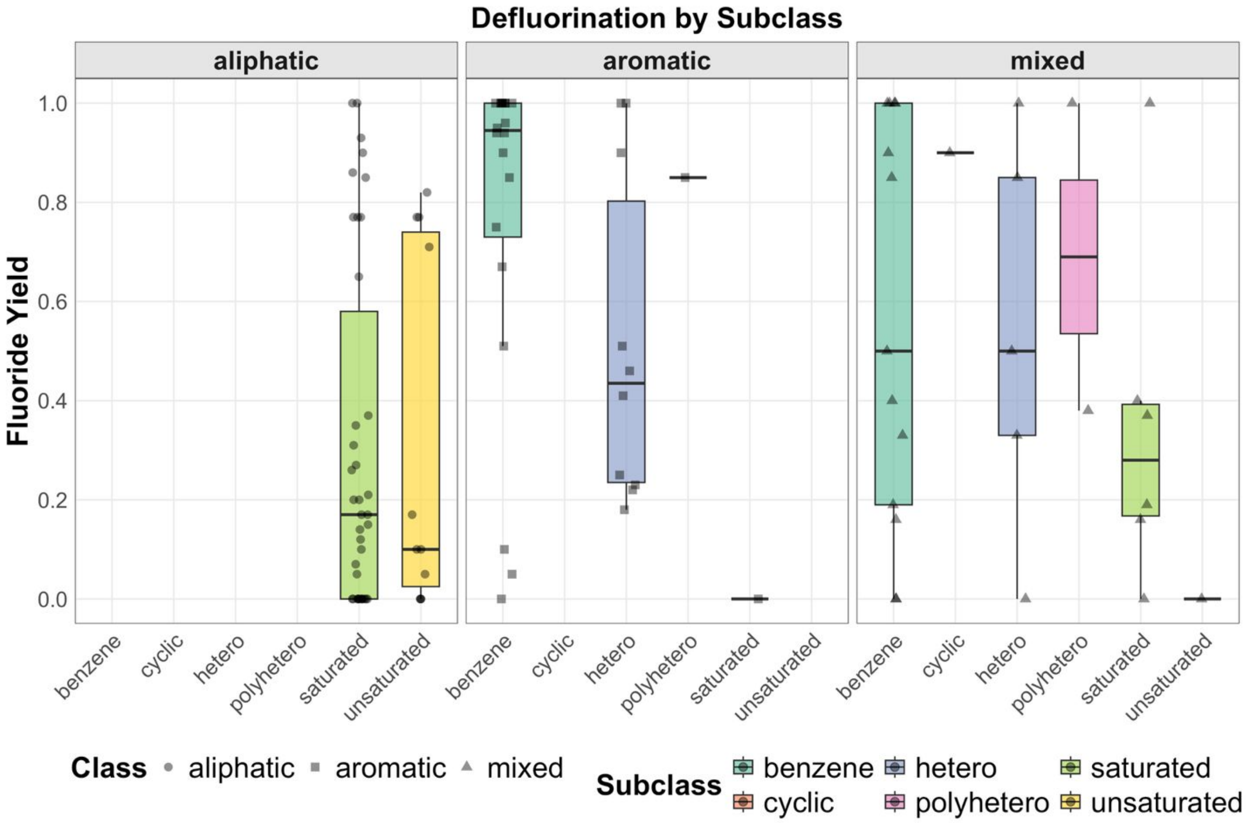

Figure S3: Fate of fluorinated molecules in the environment and correlations with molecular class and subclass. P-values for box and whisker plots (two-way ANOVA): Suclass = 0.0001, Class = 0.486, Subclass:Class = 0.538.
